# Assessing left atrial systolic contribution via pressure-volume loops in murine HFpEF

**DOI:** 10.3389/fcvm.2026.1919465

**Published:** 2026-08-18

**Authors:** Hana E. Baker, Tatyana Belle-Oladeji, Marialuisa Andreasson, Robert M. Christie, Karissa J. Hyatt, Xiaojun Wang, Barry A. Borlaug, Flora Sam

**Affiliations:** 1 Eli Lilly and Company, Indianapolis, IN, United States; 2 Department of Cardiovascular Medicine, Mayo Clinic, Rochester, MN, United States

**Keywords:** atrial systole, heart failure with preserved ejection fraction, left atrial function, left ventricular function, preclinical model, pressure-volume loop

## Abstract

Left atrial (LA) myopathy plays a critical role in the pathophysiology of heart failure with preserved ejection fraction (HFpEF). This study aimed to evaluate LA function in a murine model of hypertension-induced HFpEF, with and without obesity, by analyzing left ventricular (LV) pressure signals to quantify the contribution of LA systole to total LV volume. HFpEF was induced in C57BL/6J mice using the SAUNA model, which involves uninephrectomy, chronic aldosterone infusion, and high-salt drinking water for four weeks. To model obesity-associated HFpEF, a high-fat diet (HFD) was added to the SAUNA protocol. Invasive hemodynamic measurements were obtained, and a novel method was developed to assess atrial systole using the second-order derivative of the LV pressure signal during diastolic filling. SAUNA HFpEF mice exhibited preserved LVEF, moderate hypertension, pulmonary congestion, and diastolic dysfunction as evidenced by a prolonged time constant of relaxation (Tau). In sham control mice, atrial systole accounted for 23.0 ± 2.4% of LV volume, whereas this contribution was significantly reduced to 13.9 ± 2.3% in SAUNA HFpEF mice (*P* < 0.01). In obese sham controls, atrial systole contributed 53.1 ± 2.2%, which was markedly decreased to 36.8 ± 3.6% in obese-SAUNA HFpEF mice (*P* < 0.003). This novel method enables precise quantification of LA systolic contribution to LV filling in mice and highlights the pathophysiological effects of hypertension and obesity on LA function in HFpEF.

## Graphical Abstract

**Figure:**
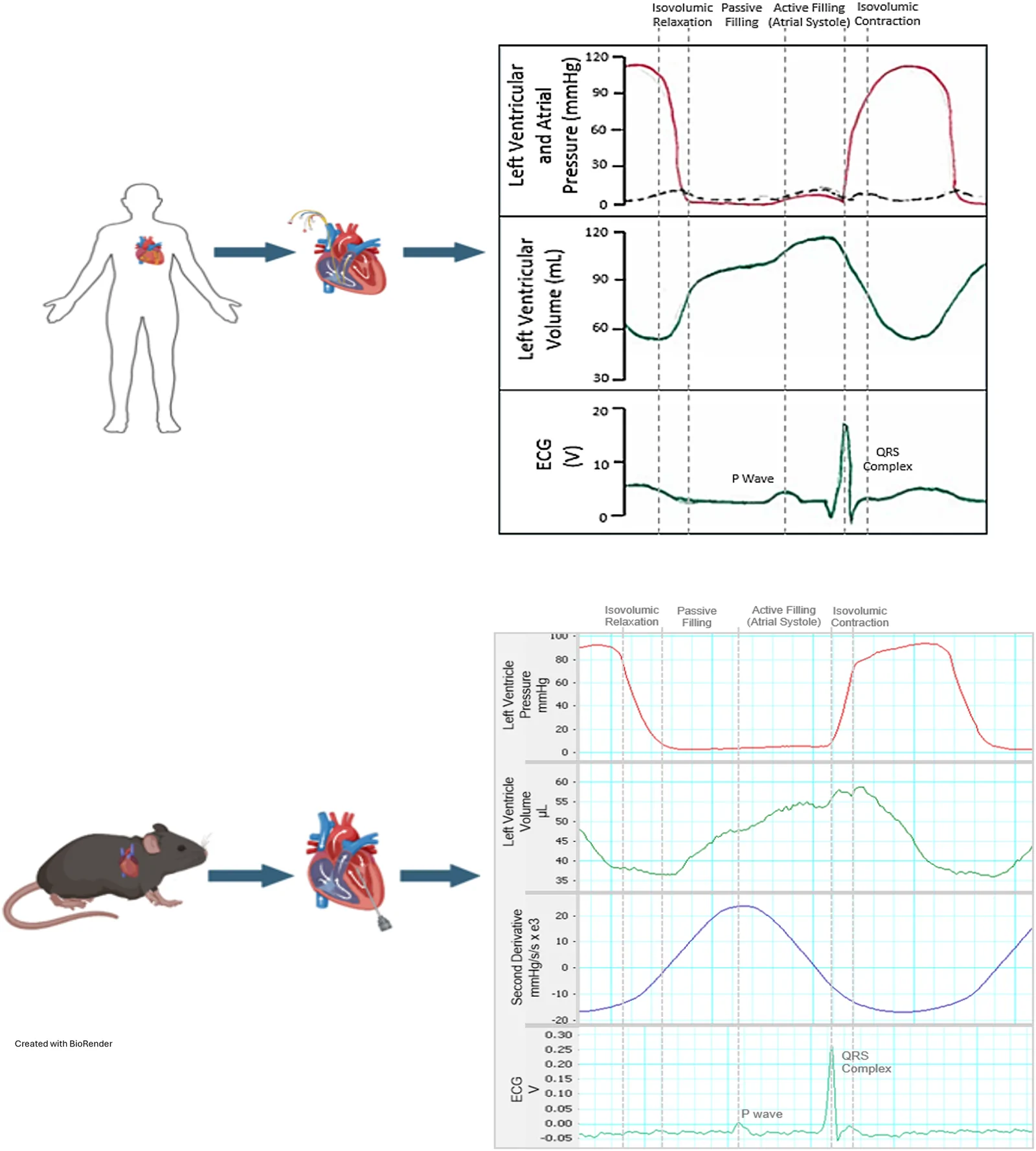
Novel method to quantify the contribution of left atrial systole to left ventricular volume in mice. Created with BioRender.

## Introduction

Heart failure (HF) remains a leading cause of morbidity and death globally, despite recent advances in therapies (1). HF with preserved ejection fraction (HFpEF), accounts for more than half of all HF cases. Although left atrial (LA) dysfunction or myopathy can occur in both HFpEF and HF with reduced ejection fraction (HFrEF), it is more prevalent in HFpEF and is associated with worse clinical outcomes (2–6). The left atrium plays a critical role in regulating left ventricular (LV) filling and maintaining cardiac output without causing pulmonary congestion. LA myopathy is a strong, independent predictor of mortality in patients with HFpEF, irrespective of LV structural or functional parameters (6). In HFpEF, LA myopathy may develop secondary to elevated LV filling pressures, in parallel with LV myopathy, or secondary to intrinsic atrial abnormalities and/or atrial fibrillation. While LA function holds diagnostic and prognostic significance in HFpEF, it remains uncertain whether improving LA function can positively influence prognosis or clinical outcomes. To address this, larger clinical studies are needed to investigate the therapeutic potential of targeting LA function (5). Similarly, there is a pressing need for improved diagnostic modalities in preclinical studies focused on LA dysfunction.

In the normal heart, the left atrium plays a pivotal role in facilitating blood flow between the pulmonary circulation and the left ventricle, functioning as a reservoir, conduit, and booster pump. Acting in concert with the mitral valve, the left atrium helps buffer pulmonary venous return from fluctuations in LV pressure. During the reservoir phase, the left atrium receives blood from the pulmonary veins during LV systole and isovolumic relaxation. In the subsequent conduit phase, blood flows passively from the left atrium into the left ventricle during LV diastole, with additional direct pulmonary venous inflow during diastasis. The final phase atrial systole or booster pump function occurs in late diastole, where active LA contraction augments LV filling by increasing end-diastolic volume, pressure, and stroke volume (7, 8). In healthy individuals, LA systole contributes approximately 20%–30% of LV stroke volume (7, 9). Consequently, impaired atrial systolic function can substantially reduce stroke volume, which is particularly detrimental in HFpEF, In this population, where LV compliance is reduced, effective LA contraction becomes crucial for maintaining adequate LV filling. Therefore, LA dysfunction in HFpEF is associated with reduced cardiac output reserve and diminished exercise capacity (2–4, 10).

Although echocardiography is considered the gold standard for assessing LA function in humans, its application in mice, commonly used as preclinical models in heart failure (HF) research, presents several challenges. These include the markedly higher heart rate, small cardiac size, and the anatomical position of the LA within the thorax, which makes obtaining conventional echocardiographic views difficult. Additionally, the need for higher imaging frame rates further complicates accurate assessment. While echocardiography remains the traditional modality for evaluating cardiac structure and function, these limitations in small rodents have rendered it suboptimal for detailed assessment of LA function (11). Alternatively, some studies have characterized atrial remodeling in experimental mouse models of heart disease by quantifying LA mass, volume, and geometric changes—chronic adaptations associated with diastolic dysfunction—and have inferred their relevance to HFpEF (12).

LA function, whether in humans or in mice, is influenced by concurrent changes in LV structure, function and composition. In this study, we present a novel method utilizing LV pressure–volume (PV) loop analysis to quantify the volume contribution of atrial systole to overall LV stroke volume in mice, serving as a functional measure of atrial performance. Evaluating LA function alongside LV contribution in murine models of HFpEF will provide a valuable diagnostic methodology for the development of targeted therapies for this challenging syndrome.

## Materials and methods

All experiments were conducted in accordance with the Guide for the Care and Use of Laboratory Animals (National Institutes of Health), and the study protocol was approved by the Indiana Institutional Animal Care and Use Committee. Male C57BL/6J mice (8–10 weeks old; Jackson Laboratories) were single-housed under a 12-hour light/dark cycle at a constant temperature of 72°F, with *ad libitum* access to standard chow (Teklad 2014; Inotiv, West Lafayette, IN) and water.

For surgical procedures, mice received a subcutaneous injection of ketoprofen (5 mg/kg) immediately prior to surgery and every 24 h thereafter for up to 72 h. Anesthesia was induced with 4%–5% isoflurane in 95% oxygen and maintained with 1%–5% isoflurane in 95% oxygen. At the conclusion of the *in vivo* experiments, mice were euthanized under anesthesia (1%–5% isoflurane in 95% oxygen) by exsanguination following heart excision. Table 1 summarizes the study groups and experimental design.

**Table 1:** Study groups, experimental design, and mapping to figures and supplementary tables.

Group	Cohort	Intervention	n	Reported in
Normal C57BL/6	Method Validation	No disease intervention (baseline PV loop)	11	Figures 2, 3; Supplementary Table S1
Sham Control	SAUNA cohort	Uninephrectomy + saline pump + normal water; standard chow	10	Figure 4; Supplementary Tables S2, S4
SAUNA HFpEF	SAUNA cohort	Uninephrectomy + aldosterone (0.35 µg/h) + 1% NaCl water, 4 wk; standard chow	11 (9 for index)	Figure 4; Supplementary Tables S2, S4
Obese Sham Control	Obese-SAUNA cohort	High-fat diet (16 wk) + sham surgery	6	Figure 5; Supplementary Tables S3, S4
Obese-SAUNA HFpEF	Obese-SAUNA cohort	High-fat diet (16 wk) + SAUNA (uninephrectomy + aldosterone + 1% NaCl, 4 wk)	6	Figure 5; Supplementary Tables S3, S4
Lean Control	Obesity-effect validation	Standard chow, no intervention	4	Supplementary Figure S2; Supplementary Table S4
Obese Control	Obesity-effect validation	High-fat diet only	4	Supplementary Figure S2; Supplementary Table S4

PV, pressure-volume; SAUNA, salt loading/uninephrectomy/aldosterone. The Lean Control and Obese Control groups were used only to characterize the effect of obesity on atrial systolic contribution (Supplementary Figure S2) and are distinct from the Obese Sham Control group of the obese-SAUNA cohort.

### HFpEF animal model

While obesity has received significant attention as a comorbidity in HFpEF, substantial evidence implicates hypertension as a primary driver of HFpEF pathogenesis. Nevertheless, hypertension is frequently overlooked in both preclinical studies and clinical practice, often relegated to a secondary role. Although obesity and type 2 diabetes mellitus (T2DM) are commonly considered equivalent contributors to HFpEF development, the condition rarely occurs in the context of obesity or T2DM without coexisting hypertension or underlying coronary artery disease (13). Therefore, we utilized the SAUNA model, a preclinical model of moderate hypertension and HFpEF, which closely reflects the clinical presentation of the disease (14, 15).

**Figure 1:**
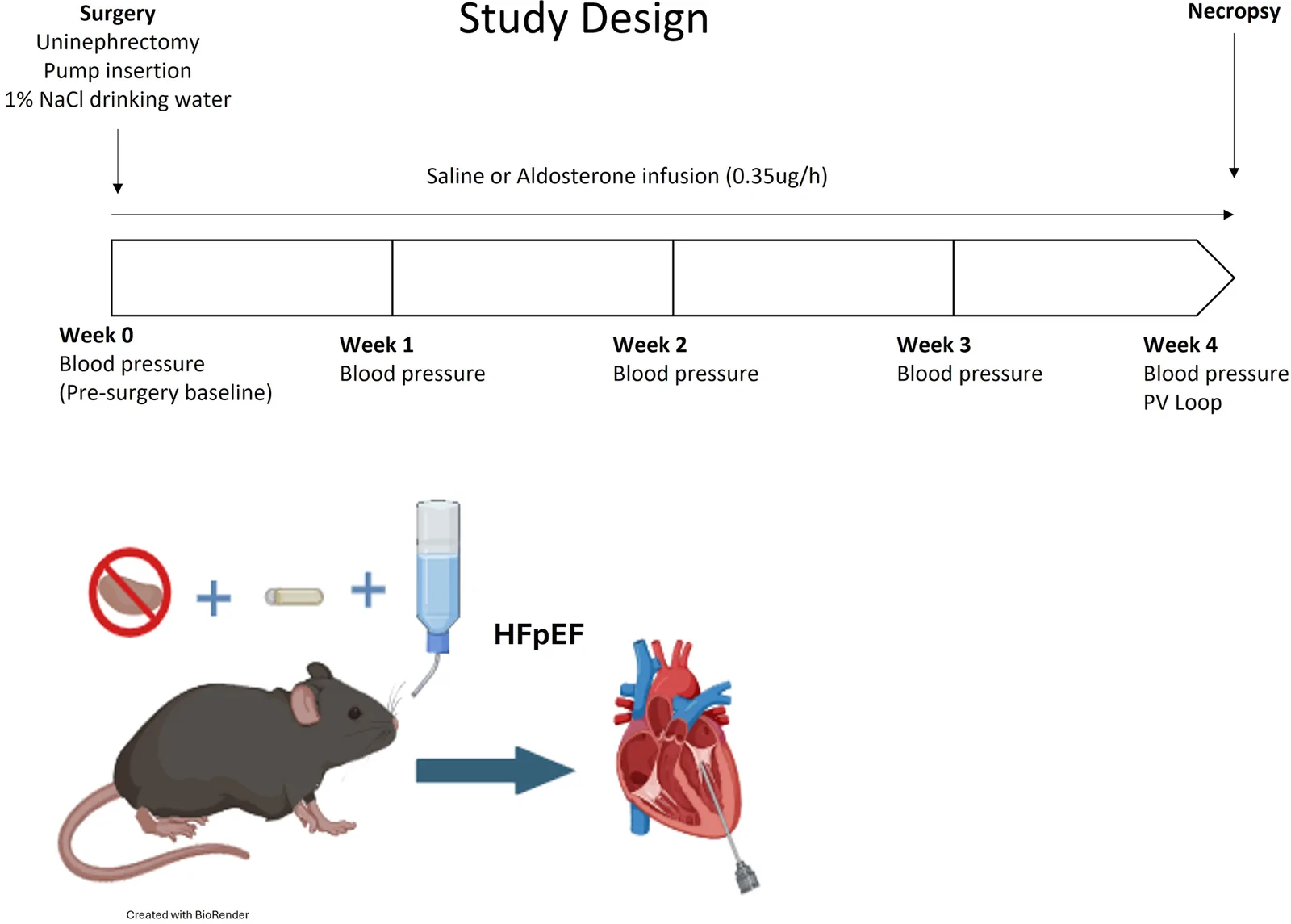
HFpEF (SAUNA) mouse study design.

The initial cohort of mice underwent either sham surgery or HFpEF induction using the SAUNA (SAlt loading/Unilateral Nephrectomy/Aldosterone infusion) model (Figure 1) (14, 15). Following ∼2-week acclimation period, and prior to HFpEF induction, mice were stratified into groups to ensure comparable distributions of body weight and blood pressure. Blood pressure was measured in conscious mice using a noninvasive tail-cuff system (Kent Scientific, Torrington, CT), after which animals were randomized using the Block Randomized Allocation Tool (BRAT; Eli Lilly and Company, Indianapolis, IN). HFpEF was induced via unilateral nephrectomy combined with continuous subcutaneous infusion of *d*-aldosterone (Sigma; A477, CAS: 52-39-1) at 0.35 µg/hr, delivered using an ALZET osmotic pump (model 1004; DURECT Corporation, Cupertino, CA), along with 1.0% sodium chloride in drinking water for four weeks, as previously described (14, 15). Sham-operated control mice underwent identical procedures; except they received saline instead of *d*-aldosterone via the osmotic pump.

### Obese-SAUNA HFpEF model

In a second cohort of mice (obese-SAUNA HFpEF), an obesogenic diet was incorporated into the SAUNA HFpEF model. Male C57BL/6J mice were fed a high-fat diet (60% kcal from fat; Teklad 06414; Inotiv, West Lafayette, IN) for 12 weeks prior to HFpEF induction and continued on the same diet for the remainder of the study (16 weeks total). This modified SAUNA protocol integrates diet-induced obesity to more closely model the metabolic comorbidities associated with HFpEF. Control mice in this cohort did not undergo SAUNA induction but were maintained on the high-fat diet for 16 weeks (obese controls).

In both cohorts, four weeks following HFpEF induction, mice underwent terminal intubation and open-chest PV loop analysis. Additionally, heart weights and lung wet/dry weights were collected at the end of the study.

### Electrocardiogram (ECG), PV catheter placement and PV analysis

All mice were anesthetized with 1.5% isoflurane in 95% O₂ and placed supine on a thermostatically controlled surgical platform (Harvard Apparatus, Holliston, MA). Once a surgical plane of anesthesia was achieved, conductive gel was applied to the digital and metacarpal pads, which were secured to the platform electrodes using surgical tape to monitor ECG signals. ECG recordings were obtained using LabChart Pro software (ADInstruments, Colorado Springs, CO).

Following ECG setup, mice were intubated via tracheotomy and mechanically ventilated (Hugo Sachs, Germany) at a tidal volume of 220 µL and a respiratory rate of 100 breaths/minute. A thoracotomy was then performed to access the heart for catheterization. A 1.2F Transonic pressure-volume catheter (Transonic Scisense, Ithaca, NY) was inserted into the left ventricle via an apical approach using a 27G needle. The catheter was advanced until the distal electrode was appropriately positioned within the LV myocardium.

Catheter placement was optimized based on the phase angle and admittance magnitude, in accordance with the manufacturer's guidelines. The phase signal was monitored to visually assess the catheter's proximity to the LV wall, and placement was adjusted to achieve the recommended minimal mean phase signal, confirming central positioning within the left ventricle. Catheter calibration was performed per the manufacturer's instructions. Steady state LV pressure and volume measurements were recorded, and PV loop data were analyzed using LabChart Pro software (ADInstruments, Colorado Springs, CO).

### Atrial systole measurements

Direct atrial pressure measurements in mice are technically challenging due to the small atrial size. Therefore, LV pressure was used as a surrogate during diastolic filling, given that LA pressure closely follows LV pressure in this phase. The onset of LA systole was identified using the second derivative of the LV pressure signal.

Using LabChart Pro software (ADInstruments, Colorado Springs, CO), the second derivative of the LV pressure was calculated at each time point by fitting a parabolic function y = a + bt + ct^2^ to the data within a defined smoothing window. The second derivative was determined as, d^2^y/dt^2^ = 2c, employing the Savitzky–Golay method (16). To enhance detection of subtle pressure changes during LV filling, the window width for the second derivative calculation was increased, allowing for the identification of abrupt shifts in the LV filling pattern indicative of LA systole onset.

A custom LabChart macro was developed to automate this process. The macro identified the peak of the LV pressure signal and the subsequent peak in its second derivative, marking the onset of atrial systole for each pressure-volume (PV) loop (see Supplementary Figure S1). It also recorded the corresponding time, pressure, and volume values.

### Quantification of atrial systolic contribution

The volume attributable to atrial systole (V_AS) was calculated as the difference between LV end-diastolic volume (EDV) and the LV volume at the onset of atrial systole (V_AS-onset). LA systolic contribution to LV filling was expressed as a percentage of LV stroke volume (SV). An integrated index was then calculated by normalizing this percentage to LV end-diastolic pressure (LVEDP), accounting for both LV preload and pulmonary venous afterload.

V_AS = EDV – V_AS-onset

LA systolic contribution (%) = (V_AS/SV) × 100

Integrated index = LA systolic contribution (%)/LVEDP

### Statistics

GraphPad Prism version 10.2.3 (GraphPad Software) was used for data visualization, calculation of group means and standard errors, and statistical analyses. Group comparisons were performed using unpaired t-tests or one-way ANOVA followed by Tukey's multiple comparisons test, as appropriate. A *p*-value < 0.05 was considered statistically significant.

## Results

### Normal mice

**Figure 2:**
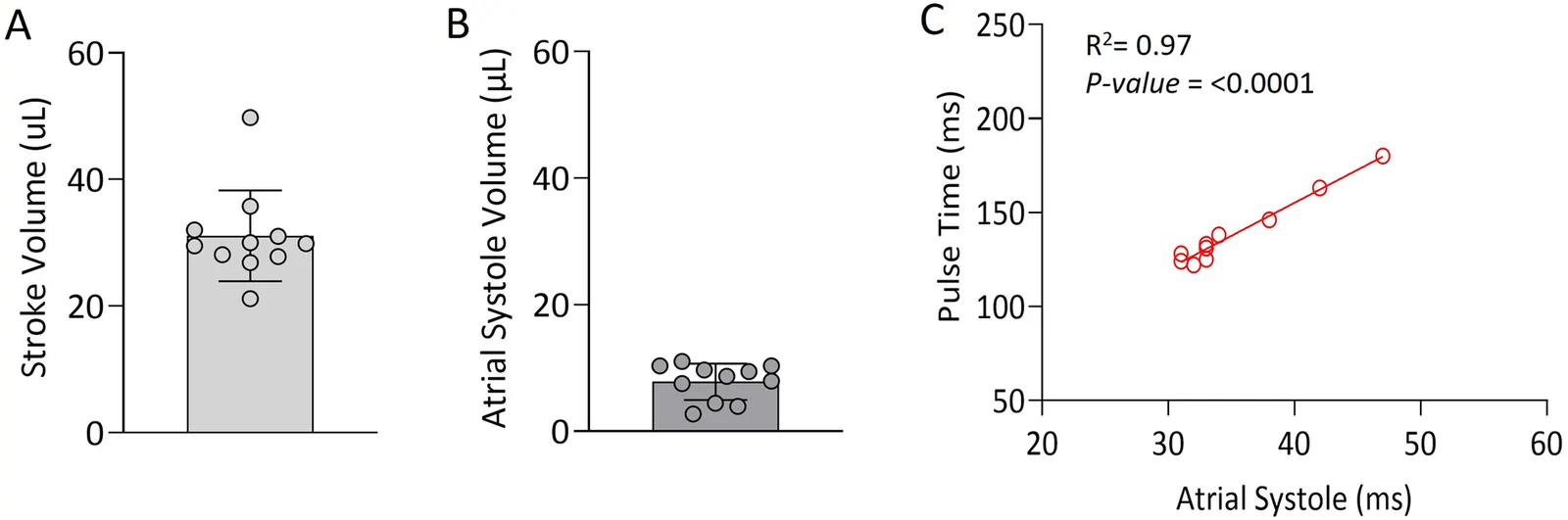
Pressure volume loop analysis in normal control mice. **(A)** Left ventricular stroke volume, **(B)** Left atrial systole volume and **(C)** the relationship between pulse time and atrial systole duration via PV loop in Sham control C57BL/6 mice (*n* = 10-11). Data are means ± SEM. Comparison by simple linear regression.

**Figure 3:**
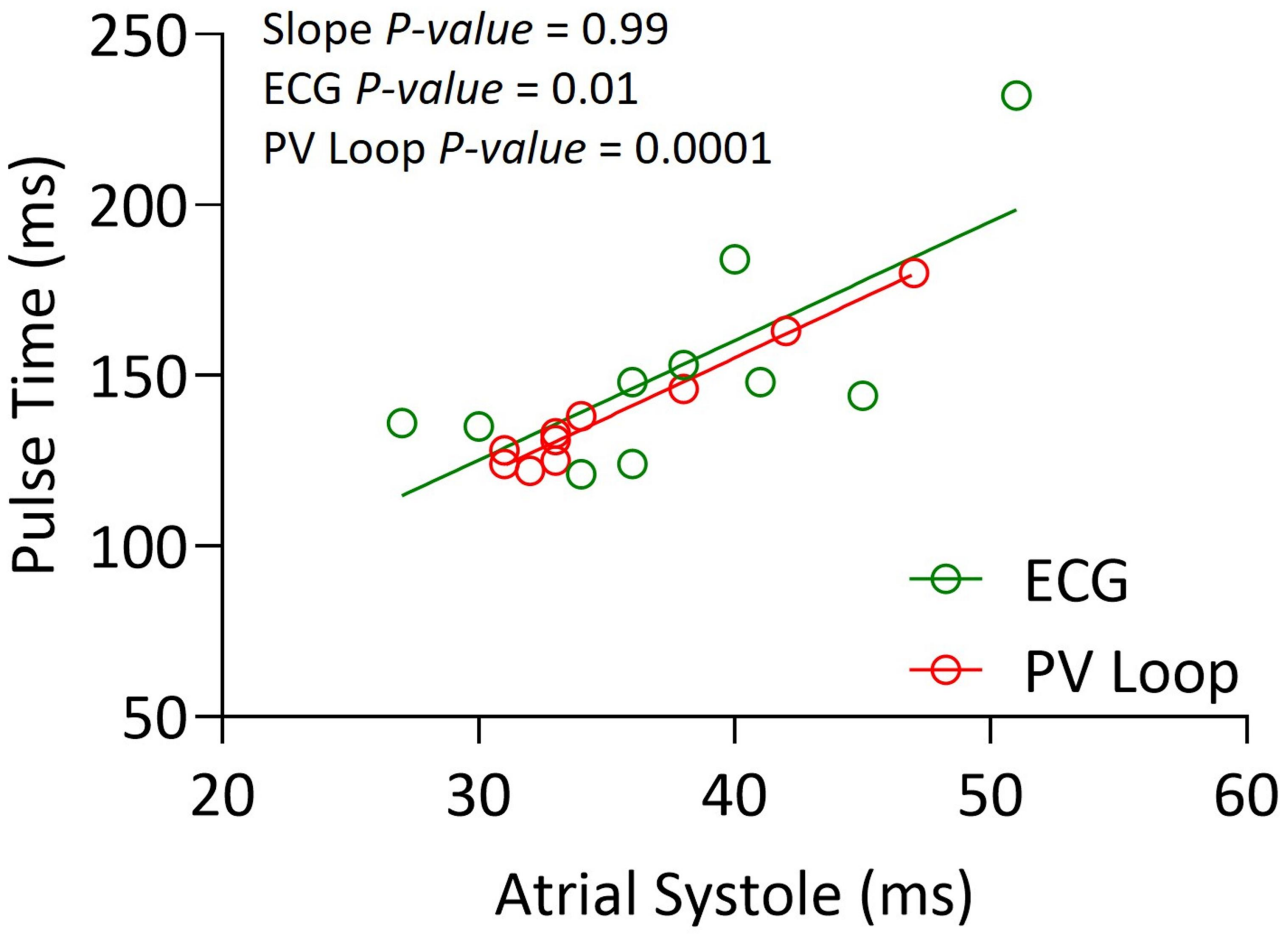
Relationship between pulse time and atrial systole duration by ECG. ECG (green open circles) and PV loop (red open circles) in normal control C57BL/6 mice (*n* = 10). Comparison by simple linear regression.

Normal C57BL/6 mice were used to validate the methodology. The mice underwent PV loop analysis to verify normal cardiac function. LV stroke volume was quantified (31.1 ± 2.2 µL) in Sham control mice, as shown in Figure 2A. The volume ejected from the left atrium into the left ventricle during atrial systole (7.8 ± 0.9 µL) is presented in Figure 2B, contributing to 25.6% of the total LV stroke volume (Supplementary Table S1). To validate the method, we evaluated the correlation between heart rate [expressed as pulse time (ms)] and atrial systole duration (ms) as determined by the second order derivative. There was a significant correlation between pulse time (ms) and atrial systole duration (ms) (*P* < 0.0001, Figure 2C) as measured by PV analysis. Furthermore, the length of atrial systole (ms) on ECG, defined as the period from the peak of the *P* wave to the start of the QRS complex, showed a significant correlation with pulse time (ms) (*P* = 0.01, Figure 3). Importantly, this relationship matched that between pulse time and atrial systole duration found in PV analysis (*P* = 0.99), as seen in Figure 3. Additionally, the peak of the *P* wave on ECG lined up with the peak of the second order derivative of the LV pressure signal (see GRAPHICAL ABSTRACT).

### SAUNA HFpEF mice

**Figure 4:**
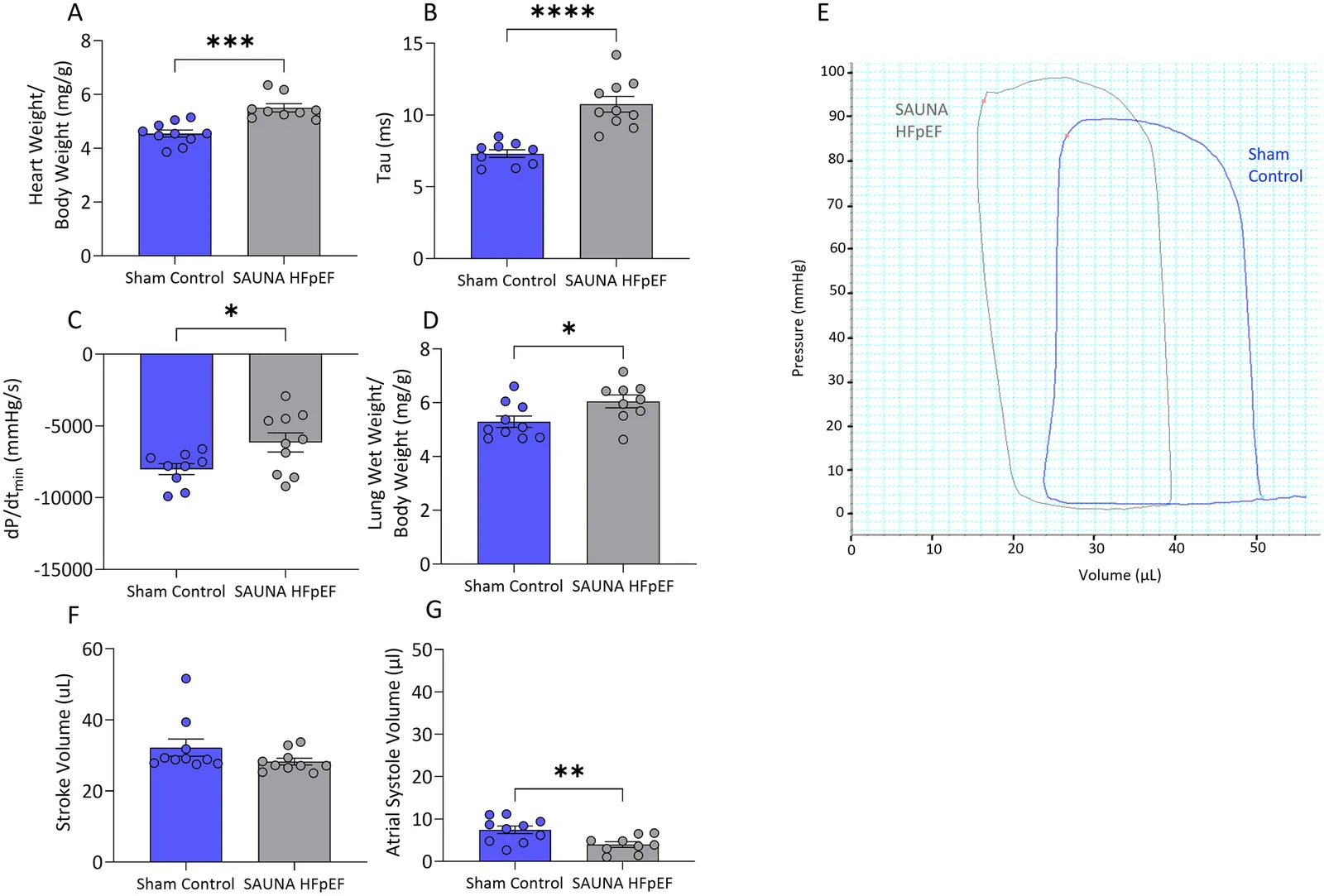
Characteristics of SAUNA HFpEF mice. **(A)**. Increased heart weight in HFpEF SAUNA mice indicative of cardiac hypertrophy. **(B)**. Pressure-volume loop analysis: HFpEF SAUNA mice demonstrated prolonged Tau indicating delayed relaxation and impaired diastolic function. **(C)** Sham control mice have a more negative dP/dt_min_ -an index of left ventricular relaxation and diastolic function- indicating better diastolic function and a quicker, more efficient relaxation process compared to HFpEF SAUNA mice. **(D)**. HFpEF SAUNA mice demonstrated increased wet lung weight (*n* = 9–10/group) **(E)**. Representative pressure-volume loops from Sham control (blue) and SAUNA HFpEF (grey) mice. **(F)**. LV stroke volume and **(G)**. Left atrial systole volume in Control sham and SAUNA HFpEF mice (*n* = 10–11/group) Data are means ± SEM. *P ≤ 0.05, ** *P* ≤ 0.01, ***P ≤ 0.0005, **** *P* ≤ 0.0001 using unpaired t-test.

Similar to prior studies (14, 15), SAUNA HFpEF mice demonstrated normal LV ejection fraction (68.4%) but exhibited significantly increased heart weight to body weight vs. Sham control mice (21% greater than Sham control, *P* < 0.0002, Figure 4A) demonstrating increased LV hypertrophy relative to Sham control mice. SAUNA HFpEF mice demonstrated diastolic dysfunction with significantly increased Tau vs. control (10.7 ± 0.5 vs. 7.3 ± 0.3 ms, *P* = 0.00004, Figure 4B) and significantly decreased dP/dt_min_ (−6152 ± 665 vs. −8014 ± 385 mmHg/s, *P* = 0.03, Figure 4C) and significantly increased wet lung weight to body weight (6.05 ± 0.24 vs. 5.29 ± 0.21 mg/g, *P* < 0.03, Figure 4D), as compared with Sham control mice. Figure 4E presents representative pressure-volume loops from both Sham control and SAUNA HFpEF mice. There was no significant difference in overall LV stroke volume between the two groups (28.3 ± 0.9 vs. 32.2 ± 2.4 µL, *P* = 0.15, HFpEF vs. Sham control Figure 4F). However, the volume received by the left ventricle during atrial systole was significantly lower in SAUNA HFpEF vs. Sham control mice (5.0 ± 0.9 vs. 7.4 ± 0.9 µL, *P* = 0.008, Figure 4G), resulting in a considerable decrease in the contribution of atrial systole to LV filling in SAUNA HFpEF vs. Sham control mice (13.9 ± 2.3 vs. 23.0 ± 2.4%, *P* = 0.01, Supplementary Table S2).

### Obese- SAUNA HFpEF mice

**Figure 5:**
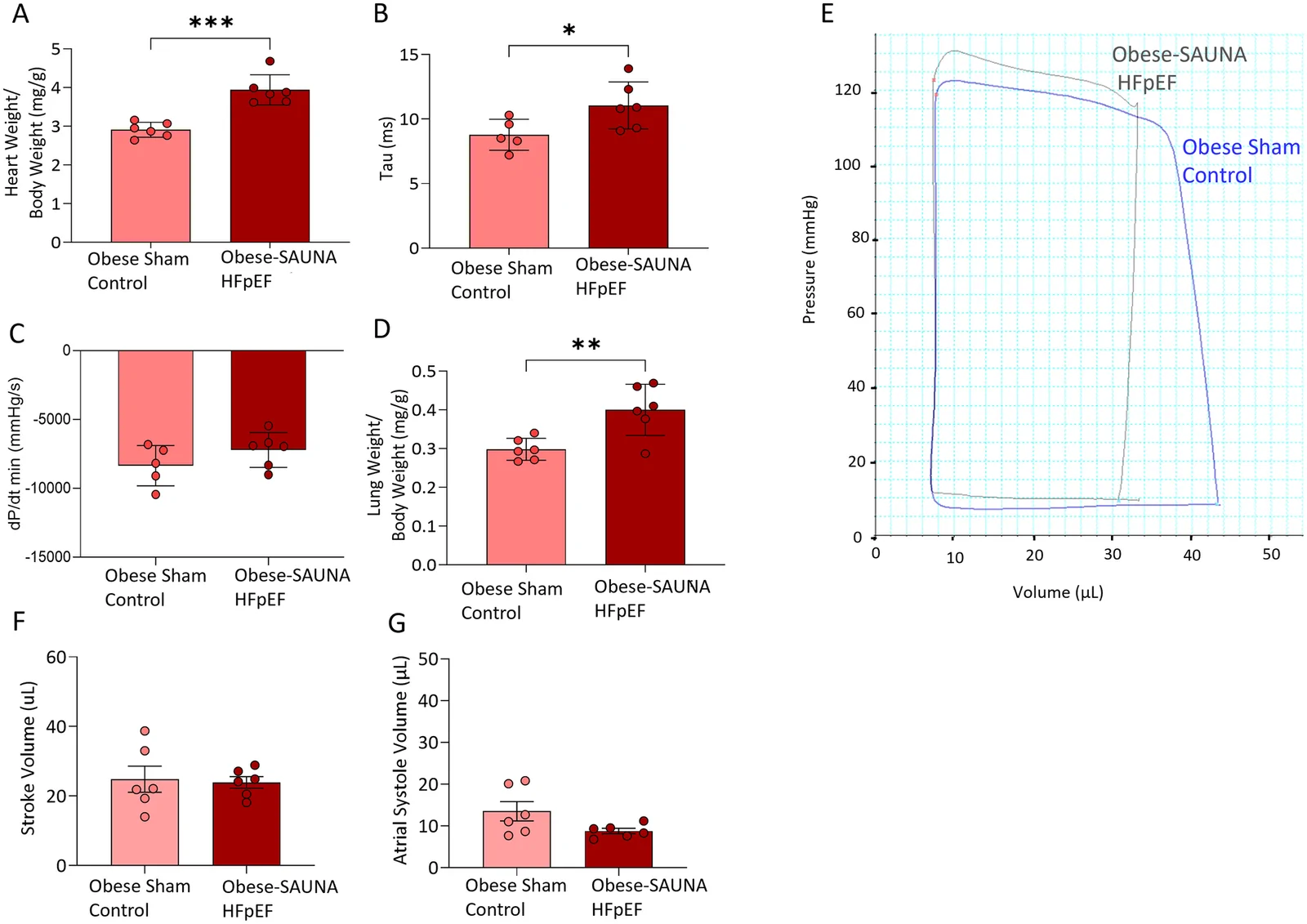
Characteristics of obese-SAUNA HFpEF mice. **(A)**. Increased heart weight in Obese-SAUNA HFpEF mice indicative of cardiac hypertrophy vs. Obese-sham **(B)**. Pressure-volume loop analysis: Obese-HFpEF SAUNA mice demonstrated prolonged Tau indicating delayed relaxation and impaired diastolic function vs. Obese-sham. **(C)**. Obese sham control mice have a more negative dP/dt_min_ and thus better diastolic function and a quicker, more efficient relaxation process compared to Obese-SAUNA HFpEF mice. **(D)**. HFpEF SAUNA mice demonstrated increased wet lung weight (*n* = 5–6/ group) **(E)**. Representative pressure-volume loops of Obese Control (Sham) (blue) and Obese-hypertension HFpEF (Grey) mice **(F)**. LV stroke volume and **(G)**. left atrial systole volume in Obese Control sham and Obese-SAUNA HFpEF mice (*n* = 6/group). Data are means ± SEM. *P ≤ 0.05, **P ≤ 0.01, ***P ≤ 0.001 using unpaired t-test.

Mice with HFpEF (SAUNA) and obesity demonstrated a HFpEF phenotype that was comparable to the original SAUNA mouse model, with preserved LV ejection fraction in obese-SAUNA HFpEF vs. obese sham control, (83.2 ± 3.1 vs. 82.1 ± 1.5%, *P* = 0.74), increased heart weight to body weight (35.4% greater than obese sham control, *P* = 0.0002, Figure 5A), prolonged Tau (11.1 ± 0.7 vs. 8.8 ± 0.5 ms, *P* = 0.04, Figure 5B) and a trend to decreased dP/dt_min_ (−7219 ± 515 vs. −8355 ± 654 vs. mmHg/s, *P* = 0.20, Figure 5C), in obese-SAUNA HFpEF vs. obese sham control. The presence of obesity induced lung congestion, evidenced by increased wet lung weight to body weight (4.00 ± 0.27 vs. 2.98 ± 0.12, *P* = 0.006, obese-SAUNA HFpEF vs. obese sham control, Figure 5D). Figure 5E demonstrates representative pressure-volume loops from both obese control and obese-SAUNA HFpEF mice. Left ventricular stroke volumes were similar between the obese sham control and obese-SAUNA HFpEF mice (23.9 ± 1.6 vs. 24.8 ± 3.8 µL, *P* = 0.83, obese-SAUNA HFpEF vs. obese sham control, Figure 5F); however, obese-SAUNA HFpEF mice had reduced blood volume entering the left ventricle during atrial systole in comparison to obese sham controls (8.8 ± 0.6 vs. 13.5 ± 2.3 µL, *P* = 0.08, Figure 5G). Therefore, the role of LA systole in contributing to total LV stroke volume was significantly reduced in the obese-SAUNA HFpEF mice compared to respective obese sham control group (36.8 ± 3.6 vs. 53.1 ± 2.2%, *P* = 0.003, obese-SAUNA HFpEF vs. obese sham control, Supplementary Table S3).

Finally, LV compliance is impaired in HFpEF and thus affects the relative LA contribution to LV end-diastolic volume, since the contribution of rapid filling and diastasis may be more prominent due to increased LA pressure and preload therefore, in both cohorts of mice, in addition to the volumetric contribution, the ratio of the % LA systole contribution to LV end-diastolic pressure (LVEDP) was determined. As seen in Supplementary Table S4, this is impaired in both SAUNA HFpEF mice and obese-SAUNA HFpEF mice vs. respective controls (5.0 ± 0.9 vs. 7.5 ± 0.6, *P* = 0.03, SAUNA HFpEF vs. Sham control and 4.4 ± 0.5 vs. 6.2 ± 0.7, *P* = 0.06, obese-SAUNA HFpEF vs. obese sham control). We propose that this integrated index accounts for “LV preload” and “pulmonary venous afterload” and demonstrates impaired LV compliance in both cohorts of mice.

## Discussion

In this study, we present a novel method to identify atrial systole from LV PV loops using the second derivative of LV pressure with respect to time. This approach enabled quantification of the LA contribution to total LV stroke volume in three groups: normal mice, SAUNA (hypertension-associated) HFpEF mice, and a combined obese-SAUNA HFpEF model. At baseline, SAUNA HFpEF mice exhibited evidence of LA dysfunction. Given that hypertension is the most prevalent comorbidity associated with HFpEF, initial assessments of LA function were performed in the SAUNA model. Furthermore, due to the strong association between obesity and hypertension—and the rising global prevalence of obesity the integration of both comorbidities into a single HFpEF mouse model enhances its clinical relevance.

Measuring LA function in rodents is essential for understanding cardiovascular physiology and pathology, particularly in conditions where cardiac structure and function are impaired. In this study, we aimed to investigate LA function as a key component of cardiac performance by leveraging the LV pressure signal to identify and quantify the volume contribution of LA systole to total LV stroke volume in HFpEF mice.

In humans and large animal models LA function is commonly assessed using echocardiography or cardiac magnetic resonance imaging (MRI) (17, 18). However, such assessments pose significant challenges in preclinical rodent studies due to the small size, unique anatomy, and positioning of the murine left atrium and appendage (19, 20). Zhang et al. proposed an innovative technique using speckle-tracking echocardiography to evaluate LA function in mice (11). They included three mouse models of atrial myopathy, one of which was an obesity-associated HFpEF model induced by a high-fat diet combined with N*ω*-nitro-L-arginine methyl ester (L-NAME). This method demonstrated impaired LA reservoir, conduit, and contractile function in HFpEF mice, as indicated by reductions in LA strain rates. Despite its innovation, this echocardiographic method remains technically challenging due to the limitations of current ultrasound probes. Specifically, the physical constraints of probe size and resolution hinder accurate quantification of left atrial volumes, further complicated by anatomical positioning and access during imaging.

Our approach directly quantifies the volume contribution of LA systole using conductance catheter data, with the LV pressure signal serving as a surrogate for LA pressure. We observed a significant reduction in LA systolic volume contribution in SAUNA HFpEF mice compared to Sham controls (Figure 5). This finding aligns with results reported by Zhang et al., who also identified impaired LA function particularly reduced LA contractile function in a distinct HFpEF model (11). While the HFpEF models differed, both models demonstrated preserved LV ejection fraction alongside evidence of LV diastolic dysfunction. In Zhang's model, this was demonstrated as elevated E/e′ and E/A ratios and reduced global longitudinal strain (GLS). In our SAUNA model, diastolic dysfunction was similarly supported by an elevated time constant of relaxation (Tau) and decreased dP/dt_min_ (Figures 4B,C). Importantly, PV loop analysis remains the gold standard for diagnosing HFpEF, providing direct insight into LV compliance and diastolic performance (21, 22).

We modified the established SAUNA protocol by incorporating a high-fat diet to introduce an obesity component, thereby highlighting obesity and hypertension. This model retains the key pathological features of the traditional SAUNA HFpEF model, including left ventricular (LV) hypertrophy, LV diastolic dysfunction, pulmonary congestion (Figure 5), and LA systolic dysfunction paralleling the clinical phenotype observed in patients with obesity-related HFpEF (23, 24). Obesity has been associated with a distinct LA functional profile, characterized by reduced reservoir and conduit function, accompanied by enhanced systolic function, which is likely representing a compensatory mechanism (25). Additionally, hypertensive heart disease is known to impair LA mechanics, including increased LA size and diminished function (26).

Consistent with human observations, our study found that obese control mice exhibited a marked increase in LA systolic volume contribution to LV filling compared to lean control mice. However, this contribution was significantly reduced in obese-SAUNA HFpEF mice, suggesting that LA contractile function declines with disease progression (Supplementary Figure S2). This apparent similarity between lean controls and obese-SAUNA HFpEF mice (Supplementary Figure S2) warrants clarification. Obesity itself elicits a compensatory increase in LA booster-bump function, such that obese control mice exhibited a markedly higher atrial systolic contribution (64.7%) than lean control mice (42.5%) (25). In obese-SAUNA HFpEF, the contribution fell to 36.8%, a decline from this elevated obese baseline toward, and slightly below, the lean level; the resemblance to lean controls therefore reflects loss of the obesity-related compensatory increment rather than preserved atrial function. Moreover, the relative percentage may remain similar while absolute measures diverge: absolute atrial systolic volume decreased from 13.5 µL in obese sham controls to 8.8 µL in obese-SAUNA HFpEF. Importantly, the physiologically appropriate comparison for each cohort is the HFpEF group vs. its own comorbidity-matched control, and atrial systolic contribution was significantly reduced in both cohorts.

Interestingly, the presence of combined comorbidities (obesity and hypertension) did not further exacerbate measures of diastolic dysfunction such as Tau or dP/dt_min_, relative to mice with hypertension alone (Supplementary Figure S5). This observation reflects clinical findings in human HFpEF, where the presence of multiple comorbidities does not necessarily correlate with worse hemodynamic parameters or clinical outcomes. Moreover, LA systolic contribution to LV filling was similarly and significantly reduced in both SAUNA HFpEF and obese-SAUNA HFpEF mice, further supporting the relevance of this combined model in mimicking human HFpEF pathophysiology.

Because the SAUNA model imposes substantial volume and pressure loading, we considered whether the reduced atrial contribution might reflect passive loading rather than intrinsic atrial dysfunction. Several observations argue against a purely passive mechanism. Increased preload would be expected to augment atrial filling via the Frank-Starling relationship, yet atrial systolic contribution was reduced in HFpEF animals. Obese controls, despite high preload, showed an enhanced atrial contribution that subsequently declined in obese-SAUNA HFpEF, a trajectory more consistent with functional deterioration than with additional volume loading. Furthermore, the integrated index normalized to LVEDP, which partially adjusts for loading, remained impaired in both cohorts. Structural atrial remodeling and fibrosis have been reported in comparable mouse HFpEF models (12), supporting an intrinsic contribution; nonetheless, direct atrial pressure and tissue measurements will be required to fully dissociated intrinsic atrial myopathy from systemic loading and ventricular remodeling.

No single clinical parameter directly quantifies the atrial systolic contribution to LV filling. Conceptually, the present metric is the invasive volumetric analogue of the LA booster-pump function assessed clinically by LA contractile strain and the transmitral A-wave/atrial filling fraction reductions in which accompany elevated E/e’ and pulmonary capillary wedge pressure, increased LA size, and higher circulating natriuretic peptide levels (7, 8, 25). We did not have access to a paired clinical dataset containing the phase-resolved volume signals needed to derive an equivalent measure; prospective validation of this metric against clinical invasive pressure-volume and echocardiographic or cardiac magnetic resonance data will therefore be an important next step to establish its translation correlates.

Finally, patients with HFpEF often present with multiple comorbidities, with hypertension and obesity being the most prevalent. However, other conditions such as atrial fibrillation (AFib), type 2 diabetes mellitus (T2DM), and obstructive sleep apnea (OSA) are also highly common. Comorbidities like OSA and T2DM can exacerbate pathological processes in individuals with AFib and atrial myopathy, creating a vicious cycle in which each condition worsens the others primarily through progressive impairment of left atrial (LA) function (27, 28). Future studies are warranted to evaluate the individual and combined contributions of these comorbidities to LA function and systolic contribution, using PV loop analysis in preclinical models of HFpEF.

### Limitations

A primary limitation of this study is that we did not directly measure LA pressure or volume; instead, these parameters were inferred from LV pressure and volume signals. Nonetheless, our approach is supported by previously published studies that used volumetric assessments to evaluate LA function in human subjects (25, 29, 30). Another limitation is the inability to concurrently assess LA systolic function with structural parameters (e.g., LA or LV dimensions), transmitral Doppler flow, or pulmonary congestion via echocardiography. This is due to the requirement for an open-chested model during PV loop acquisition. Future studies may address this by performing sequential imaging and PV loop assessments, ideally in a closed-chest model although such an approach would pose significant technical challenges. Additionally, ECG recordings could not be obtained simultaneously with PV loop data due to electrical interference. As a result, ECG assessments were conducted separately in this study. Another important limitation is the lack of a direct comparison between the SAUNA HFpEF and obese-SAUNA HFpEF models. These cohorts were studied independently. However, key parameters including Tau, dP/dt_min_, and LA systolic contribution were found to be comparable across the two models.

Moreover, the methodology presented here has not yet been validated in other disease states, such as: (i) low preload conditions, where LA contraction may have minimal effect on LV pressure; and (ii) models with stiff LV walls (e.g., restrictive cardiomyopathy), with or without severe LA dysfunction, where the atrial contribution to LV filling could be diminished or exaggerated. Finally, the study included only male mice at 10 weeks of age. Considering the higher prevalence of HFpEF in women and older adults, future studies should incorporate female and aged mice to improve the translational relevance of the findings.

Measurements were obtained using an open-chest apical approach, which can alter loading conditions by reducing preload and afterload and eliminating the normal pericardial constraint. These effects may have blunted hemodynamic differences between groups and likely contributed to the similar LV end-diastolic pressures observed. An additional limitation is the measurement of LV end-diastolic pressure (LVEDP) itself. In mice, high and variable heart rates markedly shorten diastole, making it difficult to identify a discrete and stable end-diastolic point. Beat-to-beat respiratory variation, the open-chest preparation, and sensitivity to catheter position and calibration further complicate accurate LVEDP measurement from pressure–volume loops (19). Because murine LVEDP values are typically low and influenced by heart rate and anesthetic depth, LVEDP may be relatively insensitive for detecting diastolic dysfunction under these conditions. We therefore interpret the modest filling pressures as reflecting the open-chest preparation and elevated heart rates rather than the absence of an HFpEF phenotype, particularly given the accompanying LV hypertrophy, prolonged Tau, reduced dP/dt_min_, and pulmonary congestion. Although a closed-chest retrograde carotid approach is more physiological, it is technically demanding; thus, closed-chest validation remains an important priority for future studies.

We also did not directly measure chamber stiffness (e.g., the end-diastolic pressure-volume relationship); because the second derivative of the LV pressure signal is used to identify the timing of atrial systole rather than to quantify atrial force, increased ventricular stiffness is unlikely to spuriously inflate the volumetric contribution, but timing detection in markedly stiff ventricles warrants further validation. Finally, the SAUNA model was selected because hypertension is the most prevalent comorbidity in clinical HFpEF and the model produces moderate hypertension with preserved ejection fraction that closely reflects the clinical phenotype (13–15); alternative models such as the L-NAME/high-fat-diet “two-hit” model emphasize combined metabolic stress and nitric-oxide-synthase inhibition and could be examined in the future applications of this method.

### Conclusion

LA myopathy plays a critical role in the pathophysiology of HFpEF. In this study, we present a novel method to quantify the volume contribution of atrial systole to overall left ventricular (LV) stroke volume in mice using LV pressure-volume loop analysis. Our findings demonstrate that LA systolic function, as reflected by atrial volume transfer, is significantly impaired in both SAUNA HFpEF and obese-SAUNA HFpEF mouse models. Interestingly, the obese-SAUNA model initially exhibited enhanced LA systolic contribution, likely due to obesity-related hyperfunction, which subsequently declined as the disease progressed to overt obese-hypertensive HFpEF. This observation supports a dynamic trajectory of LA function in the context of multiple comorbidities. The application of this technique offers a new tool for investigating the pathophysiological mechanisms of LA dysfunction and may inform therapeutic strategies targeting atrial function in preclinical models of HFpEF and other atrial myopathic conditions. Further studies are warranted to explore its broader applicability and translational relevance.

## Data Availability

The original contributions presented in the study are included in the article/Supplementary Material, further inquiries can be directed to the corresponding author.
