# Supplementary material for: Assessing left atrial systolic contribution via pressure-volume loops in murine HFpEF
Source: Front Cardiovasc Med. 2026 Aug 18;13:1919465. doi: 10.3389/fcvm.2026.1919465 (PMC13529998; doi:10.3389/fcvm.2026.1919465)
Supplement: Supplementary file 1 [file Datasheet1.docx]

**Supplementary Data**

**Assessing Left Atrial Systolic Contribution via Pressure-Volume Loops in Murine HFpEF**

Hana E. Baker^1^, Tatyana Belle-Oladeji^1^, Marialuisa Andreasson^1^, Robert M. Christie^1^,

Karissa J. Hyatt^1^, Xiaojun Wang^1^, Barry A. Borlaug^2^, Flora Sam^1^

^1^Eli Lilly and Company, Indianapolis, IN, USA

^2^Department of Cardiovascular Medicine, Mayo Clinic, Rochester, MN, USA

**Running title:** Left Atrial Function via PV Loop in HFpEF Mice

**Word count:** 1047

**Corresponding Author:**

Dr Hana E. Baker

Cardiometabolic Research (CMR)

Eli Lilly and Company

Lilly Corporate Center, Indianapolis, IN, 46285 USA

Telephone: +1 317.655.8867

Email: baker_hana_e@lilly.com

**Supplemental Figure 1.** Macro for identification of atrial systole

**Supplemental Figure 2.** Atrial systole contribution to left ventricular filling in Lean Control (42.5 ± 2.5%), Obese Control (64.7 ± 1.2%), Obese Sham Control (53.1 ± 2.2%), and Obese-Hypertensive HFpEF (36.8 ± 3.6%). Lean Control vs. Obese Control (P = 0.0007), Obese Control vs. Obese-Hypertensive HFpEF (P < 0.0001) and Obese Sham Control vs. Obese-Hypertensive HFpEF (P = 0.0019). Data are means ± SEM. **P ≤ 0.01, *** P ≤ 0.001 and **** P ≤ 0.0001 using one-way ANOVA with Tukey’s multiple comparison test.

**Supplemental Table 1.**

Cardiac contractile function in normal C57BL/6 mice

| Group | Stroke Work (mmHg*µL) | Cardiac Output (µL/min) | Stroke Volume (µL) | Atrial Systole Volume (µL) | Atrial Systole (%) | End Systolic Volume (µL) | Beginning Atrial Systolic Volume (µL) | End Diastolic Volume (µL) | End Systolic Pressure (mmHg) | End Diastolic Pressure (mmHg) | Heart Rate (bpm) | Ejection Fraction (%) | dP/dt max (mmHg/s) | dP/dt min (mmHg/s) | Tau (ms) |
| --- | --- | --- | --- | --- | --- | --- | --- | --- | --- | --- | --- | --- | --- | --- | --- |
| C57BL/6 | 2092 ± 157 | 13172 ± 1343 | 31.1 ± 2.2 | 7.8 ± 0.9 | 25.6 ± 2.9 | 33.7 ± 5.1 | 54.4 ± 6.7 | 64.8 ± 6.9 | 82.3 ± 5.9 | 3.9 ± 0.3 | 421 ± 22 | 51.6 ± 3.1 | 8156 ± 841 | -7213 ± 860 | 7.9 ± 0.7 |
| Pacher et al. | 1200-2700 | 7000-16000 | 14-30 | N/A | N/A | 2-21 | N/A | 20-53 | 80-120 | 1-8 | 470-655 | 55-72 | 8200-16000 | 6000-12000 | 7-12 |

Values are mean ± SEM for C57BL/6 (n = 11).

**Supplemental Table 2.**

Cardiac contractile function in SAUNA HFpEF mice

| Group | Stroke Work (mmHg*µL) | Cardiac Output (µL/min) | Stroke Volume (µL) | Atrial Systole Volume (µL) | Atrial Systole (%) | End Systolic Volume (µL) | Beginning Atrial Systolic Volume (µL) | End Diastolic Volume (µL) | End Systolic Pressure (mmHg) | End Diastolic Pressure (mmHg) | Heart Rate (bpm) | Ejection Fraction (%) | dP/dt max (mmHg/s) | dP/dt min (mmHg/s) | Tau (ms) |
| --- | --- | --- | --- | --- | --- | --- | --- | --- | --- | --- | --- | --- | --- | --- | --- |
| Sham Control | 2714 ± 207 | 14010 ± 1164 | 32.2 ± 2.4 | 7.4 **±** 0.9 | 23.0 ± 2.4 | 20.1 ± 3.7 | 44.9 ± 4.9 | 52.3 ± 5.6 | 84.5 ± 2.4 | 3.0 ± 0.2 | 434 ± 10 | 63.9 ± 3.7 | 7756 ± 396 | -8014 ± 385 | 7.3 ± 0.3 |
| HFpEF | 2822 ± 252 | 10604 ± 480 | 28.3 ± 0.9 | 5.0 ± 0.9* | 13.9 ± 2.3* | 13.5 ± 1.4 | 38.1 ± 1.6 | 41.8 ± 1.9 | 98.6 ± 6.5 | 3.0 ± 0.3 | 374 ± 8 | 68.4 ± 2.3 | 8806 ± 601 | -6152 ± 665* | 10.7 ± 0.5* |

Values are mean ± SEM for Sham Control (n = 10) and SAUNA HFpEF (n = 11). Statistical comparison by unpaired t-test. * *P* < 0.05 vs. Control.

**Supplemental Table 3.**

Cardiac contractile function in Obese-Hypertensive HFpEF mice

| Group | Stroke Work (mmHg*µL) | Cardiac Output (µL/min) | Stroke Volume (µL) | Atrial Systole Volume (µL) | Atrial Systole (%) | End Systolic Volume (µL) | Beginning Atrial Systolic Volume (µL) | End Diastolic Volume (µL) | End Systolic Pressure (mmHg) | End Diastolic Pressure (mmHg) | Heart Rate (bpm) | Ejection Fraction (%) | dP/dt max (mmHg/s) | dP/dt min (mmHg/s) | Tau (ms) |
| --- | --- | --- | --- | --- | --- | --- | --- | --- | --- | --- | --- | --- | --- | --- | --- |
| Obese Sham Control | 2720 ± 423 | 11664 ± 1902 | 24.8 ± 3.8 | 13.5 ± 2.3 | 53.1 ± 2.2 | 5.3 ± 0.6 | 16.7 ± 2.2 | 30.2 ± 4.3 | 109.4 ± 3.1 | 9.1 ± 1.1 | 469 ± 24 | 82.1 ± 1.5 | 10776 ± 292 | -8355 ± 654 | 8.8 ± 0.5 |
| Obese HFpEF | 2713 ± 151 | 9664 ± 516 | 23.9 ± 1.6 | 8.8 ± 0.6 | 36.8 ± 3.6* | 5.0 ± 1.2 | 20.1 ± 2.2 | 29 ± 2.3 | 114.1 ± 3.1 | 8.5 ± 0.3 | 408 ± 21 | 83.2 ± 3.1 | 11109 ± 765 | -7219 ± 515 | 11.1 ± 0.7* |

Values are mean ± SEM for Obese Sham Control (n = 6) and Obese-Hypertensive HFpEF (n = 6). Statistical comparison by unpaired t-test. * *P* < 0.05 vs. Obese Sham Control.

**Supplemental Table 4**

Ratio of the % LA systole contribution to LV end-diastolic pressure (LVEDP).

| Group | Atrial Systole (%)/End Diastolic Pressure (mmHg) |
| --- | --- |
| Normal C57BL/6 | 6.9 ± 0.9 |
|  |  |
| Sham Control | 7.5 ± 0.6 |
| SAUNA HFpEF | 5.0 ± 0.9* |
|  |  |
| Obese Sham Control | 6.2 ± 0.7 |
| Obese-SAUNA HFpEF | 4.4 ± 0.5 |
|  |  |
| Lean Control | 5.0 ± 1.0 |
| Obese Control | 6.6 ± 1.3 |

Values are mean ± SEM for Normal C57BL/6 (n = 11), Sham Control (n= 10), SAUNA HFpEF (n = 9), Obese Sham Control (n = 6), Obese-Hypertensive HFpEF (n = 6), Lean Control (n = 4) and Obese Control (n = 4). Statistical comparison by unpaired t-test. * *P* < 0.05 vs. respective control.

**Supplemental Table 5.**

**Side-by-side comparison of the SAUNA and obese-SAUNA HFpEF models and their respective controls.**

| Parameter | Sham Control | SAUNA HFpEF | Obese Sham Control | Obese-SAUNA HFpEF |
| --- | --- | --- | --- | --- |
| n | 10 | 11 | 6 | 6 |
| Stroke volume (µL) | 32.2 ± 2.4 | 28.3 ± 0.9 | 24.8 ± 3.8 | 23.9 ± 1.6 |
| Atrial systole volume (µL) | 7.4 ± 0.9 | 5.0 ± 0.9* | 13.5 ± 2.3 | 8.8 ± 0.6 |
| Atrial systole (%) | 23.0 ± 2.4 | 13.9 ± 2.3* | 53.1 ± 2.2 | 36.8 ± 3.6* |
| Integrated index (% / LVEDP) | 7.5 ± 0.6 | 5.0 ± 0.9* | 6.2 ± 0.7 | 4.4 ± 0.5 |
| Ejection fraction (%) | 63.9 ± 3.7 | 68.4 ± 2.3 | 82.1 ± 1.5 | 83.2 ± 3.1 |
| End-diastolic pressure (mmHg) | 3.0 ± 0.2 | 3.0 ± 0.3 | 9.1 ± 1.1 | 8.5 ± 0.3 |
| End-systolic pressure (mmHg) | 84.5 ± 2.4 | 98.6 ± 6.5 | 109.4 ± 3.1 | 114.1 ± 3.1 |
| dP/dt max (mmHg/s) | 7756 ± 396 | 8806 ± 601 | 10776 ± 292 | 11109 ± 765 |
| dP/dt min (mmHg/s) | -8014 ± 385 | -6152 ± 665* | -8355 ± 654 | -7219 ± 515 |
| Tau (ms) | 7.3 ± 0.3 | 10.7 ± 0.5* | 8.8 ± 0.5 | 11.1 ± 0.7* |
| Heart rate (bpm) | 434 ± 10 | 374 ± 8 | 469 ± 24 | 408 ± 21 |

Values are mean ± SEM. The SAUNA and obese-SAUNA cohorts were studied independently; accordingly, this comparison is presented as descriptive and no statistical tests were performed between cohorts. Asterisks (*) denote a significant difference (P < 0.05) versus the respective within-cohort control, as reported in Supplemental Tables 2–4. LVEDP, LV end-diastolic pressure.
